# Sarcopenia and frailty in critical illness

**DOI:** 10.1097/MCO.0000000000001123

**Published:** 2025-03-12

**Authors:** Marielle Johanna Maria Magdalena van der Steen-Dieperink, Wilhelmina Aria Christina Koekkoek, Imre Willemijn Kehinde Kouw

**Affiliations:** a Department of Intensive Care Medicine, Martini Hospital, Groningen; b Division of Human Nutrition and Health, Wageningen University & Research, Wageningen,; c Department of Intensive Care, Gelderse Vallei Hospital, Ede, The Netherlands

**Keywords:** frailty, mobilization, nutrition, quality of life, sarcopenia

## Abstract

**Purpose of review:**

Sarcopenia and frailty are common conditions, associated with worse clinical outcomes during critical illness. Recent studies on sarcopenia and frailty in ICU patients are presented in this review, aiming to identify accurate diagnostic tools, investigate the effects on clinical and functional outcomes, and propose possible effective interventions.

**Recent findings:**

The recent change of the sarcopenia definition underlines the importance of muscle strength over mass, this is however challenging to assess in ICU patients. There is currently no unified sarcopenia definition, nor standard frailty assessment tool; Clinical Frailty Scale is most frequently used in the ICU. Meta-analyses show worse clinical and functional outcomes for frail as well as sarcopenic patients admitted to the ICU, regardless of admission diagnosis. Frailty is a dynamic condition, worsening in severity by the time of hospital discharge, but showing improvement by 6 months post-ICU. Therapeutic interventions for frailty and sarcopenia remain limited. Although mobilization strategies show promise in improving functional and cognitive outcomes, inconsistent outcomes are reported. Heterogeneity in definitions, patient populations, and care practices challenge interpretation and comparison of study results and recognition of beneficial interventions. This highlights the need for more research.

**Summary:**

The importance of preexisting sarcopenia and frailty is recognized in ICU patients and associated with worse clinical outcomes. Multidimensional interventions are most promising, including patient-tailored mobilization and nutrition.

## INTRODUCTION

With the increase in the aging population there is an increasing number in older critical care patients. Older patients admitted to the intensive care unit (ICU), frequently present with conditions such as sarcopenia and frailty. These conditions, marked by reduced physiological reserves and muscle function, are highly prevalent among critically ill patients, with sarcopenia affecting up to ∼40% and frailty up to ∼30% of this population [1]. Although these conditions were traditionally linked to the geriatric population, sarcopenia and frailty are not exclusive to the older critical care patients but also affect the younger population. Sarcopenia and frailty are further compounded during prolonged ICU stay, with a significant amount of muscle mass loss occurring within the first week of ICU admission and persistent neuromuscular impairments, referred to as ICU-acquired weakness, lasting months to years post-ICU discharge [2].

Many patients enter critical care in a preexisting state of sarcopenia or frailty. This review aims to explore the definitions, clinical implications, and potential interventions of this group of ICU patients with sarcopenia and/or frailty, focusing on strategies to mitigate their impact in critical care settings and the recovery after critical illness. 

**Box 1:**
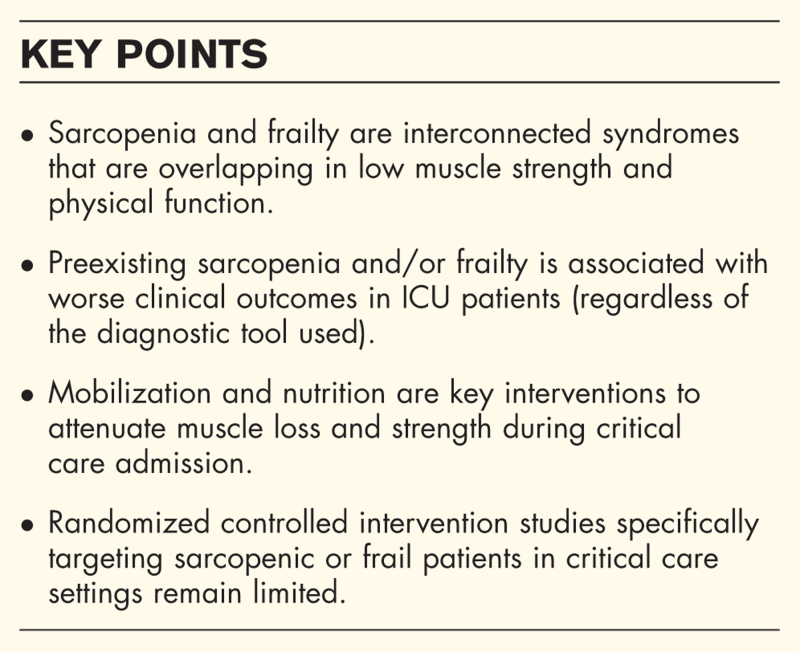
no caption available

## DEFINITIONS OF SARCOPENIA AND FRAILTY IN ICU PATIENTS

Sarcopenia and frailty are interconnected syndromes associated with aging and weakness, yet their definitions remain a subject of debate, leading to heterogeneity in patient populations and study outcomes [3–7].

### Sarcopenia

Sarcopenia has traditionally been defined as the loss of (skeletal) muscle mass and strength. The Sarcopenia Definitions and Outcomes Consortium (SDOC) redefined sarcopenia in 2020, emphasizing low muscle strength and poor function, assessed by grip strength and gait speed, as better predictors of outcomes than muscle mass. However, assessing sarcopenia in ICU patients is challenging due to limitations in measuring parameters like muscle strength and physical performance. Traditionally considered a geriatric syndrome, sarcopenia also occurs in younger, inactive individuals and those with conditions such as sarcopenic obesity, which combines obesity with impaired skeletal muscle mass and strength [8].

### Frailty

Frailty is a multidimensional and dynamic condition characterized by declines in reserve and function across multiple physiological, psychological, as well as social systems, compromising the ability to cope with every day or acute stressors [3]. It manifests heterogeneously, influenced by factors such as age, gender, comorbidities, and compensatory strategies that mask its impact in daily life. Critical illness often strips away these compensatory mechanisms, making frailty more visible. Emerging concepts such as intrinsic capacity (a holistic view of physiological reserves proposed by the World Health Organization) and resilience (focusing on an individual's ability to recover from stressors) provide additional perspectives on frailty's dynamic nature [7].

Interestingly, gender differences may also play a role in resilience regardless of frailty severity. In a posthoc analysis of two multicenter studies on 7555 very elderly patients (>80 years) admitted to the ICU, comparing baseline characteristics, male patients were significantly younger and less often frail, but had higher SOFA-scores. After propensity score matching, correcting for baseline differences, male sex was associated with higher 30-day-mortality [9].

### A dynamic process

**FIGURE 1:**
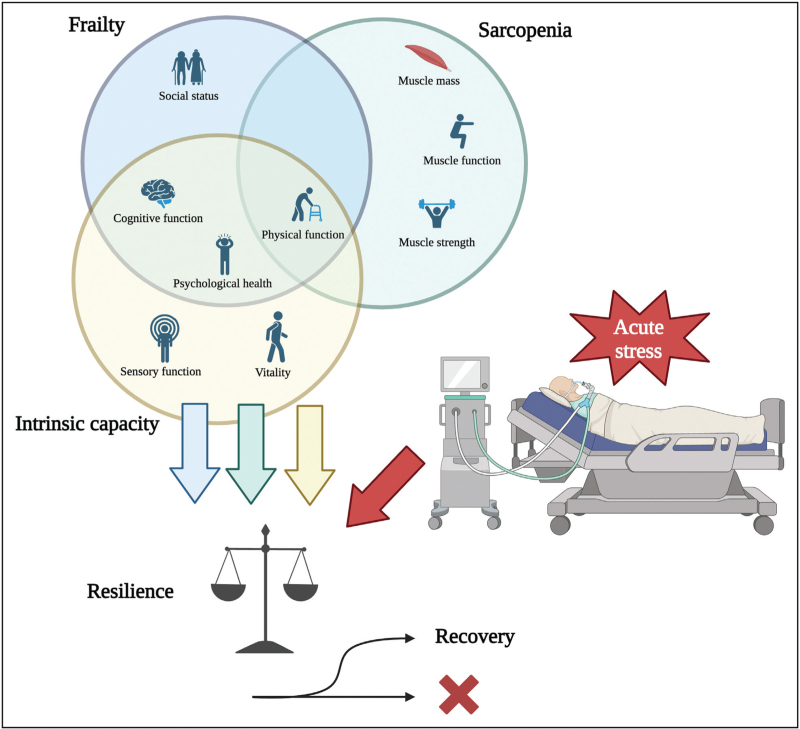
Multidimensional framework of frailty and sarcopenia highlighting the interconnected aspects of the physical factors. An individuals’ intrinsic capacity and resilience in response to acute stress will affect clinical outcomes and recovery after critical illness. Figure created in https://BioRender.com.

Both sarcopenia and frailty are dynamic conditions that may predate or develop during ICU stays, emphasizing the need for early recognition and consistent monitoring in critical care patients (Fig. 1).

## DIAGNOSTIC TOOLS FOR SARCOPENIA AND FRAILTY ASSESSMENT

The urgence for sarcopenia and frailty assessment in the acute stages of critical illness is underlined by the impact on clinical outcomes and overall survival, which necessitates the evaluation of burdensome intensive care treatments for an individual patient based upon preadmission physical status. Moreover, frailty assessment may serve as one amongst several tools for informed decision-making in sensible decisions and situations.

For sarcopenia the assessment of skeletal muscle mass, muscle strength, and physical performance are key parameters. Accurate assessment of skeletal muscle mass is challenging in the ICU; the use of ‘gold standard’ imaging methods such as magnetic resonance imaging (MRI), computed tomography (CT; arms, legs or the 3^rd^ lumbar region), and dual X-ray absorptiometry (DXA) are not always feasible in critically ill patients. Ultrasonography and bio-electrical impedance (BIA) have gained momentum as bedside alternatives to determine skeletal muscle thickness, cross-sectional area (CSA), or fat free mass, but these techniques rely on estimations and assumptions and are influenced by differences in methodology and patient variations that are difficult to control in a clinical setting (e.g. physical activity level, nutritional intake, and fluid status). While these techniques are more commonly used and feasible in the ICU, there is no consensus however on the validity of these techniques for sarcopenia diagnosis in critically ill patients. For skeletal muscle strength, dynamometer handgrip strength or isometric torque methods are frequently used in clinical practice. While physical performance and muscle function can also be evaluated through gait speed tests (e.g., 6-meter walking test), sit-to-stand tests, or the short physical performance battery, these assessments are often not feasible in critically ill patients.

Two prominent frailty assessment conceptualizations include the (Fried's) frailty phenotype (FP) [10] and the (Canadian Study of Health and Aging's, CHSA) frailty index (FI, e.g. deficit accumulation) [11]. The FP hypothesizes that frailty has an underlying biological basis that leads to five physical symptoms such as low hand grip strength, self-reported exhaustion, slowed walking speed, low physical activity, and/or unintentional weight loss; any three of which mark a person as recognizably frail. The FP is rather unidimensional, focusing on the physical domain of functioning, including a triad of frailty, malnutrition, and sarcopenia. A more multidimensional approach, the FI, hypothesizes that frailty is driven by a number of physical and psychosocial and medical deficits accumulated over time including disability, diseases, physical and cognitive impairment, psychosocial risk factors, and geriatric syndromes. The more deficits someone has, the less likely he or she is to be able to tolerate additional deficits and is therefore more likely to be frail. The FI is calculated as a ratio of the number of health deficits present in a patient divided by the total number of possible clinical deficits, derived from a representative 5-year prospective cohort study (CHSA). The FI was further developed in a more efficient and clinical applicable assessment tool based upon clinical judgement, the Clinical Frailty Scale (CFS) [12], which is based on a model of fitness, function, and independence in daily life comprising seven categories of frailty ranging from very fit (robust health) to severely frail (complete functional independence). Additionally, there are several less common instruments such as Groningen Frailty Indicator [13], Morley FRAIL scale [14], and shorter and simplified versions of the FI, such as the modified frailty index (mFI-5 and 11) [15], and CFS, simplified CFS and modified eight-category CFS [12]. In addition to frailty assessment tools, other widely used instruments to assess a person's independence in common activities of daily living, physical, and cognitive performance, and quality of life assessment are available.

A recent systematic review by Bertschi *et al.*[16] evaluated the standards for frailty assessment in the ICU, showing 19 different methods with CFS being most frequently used (∼60%). Cut-offs, time points, and manner of baseline assessment of frailty assessment upon ICU admission varied widely and only ∼40% of the studies assessed functional status. In a recent review, including 26 studies, CFS was found to be an independent predictor after an acute illness both of 30 day and 6 month-mortality [17]. Muscle function seems to be an important determinant of clinical outcome during critical illness. Nonetheless, skeletal muscle mass upon ICU admission and the loss during ICU stay has been associated with poor clinical outcomes post-ICU. Frailty at ICU admission is independently associated with leg muscle CSA loss during critical illness [18] and associated with increased mortality [19]. In a prospective study in 120 patients, Quadriceps Muscle Layer Thickness (QMLT) decreased progressively with higher CFS scores [19]. While there is currently no unified standard for assessing muscle function as part of sarcopenia or frailty in the ICU, the assessment of muscle mass is likely most feasible and of clinical relevance, especially since muscle strength and function measurements are challenging to conduct in critically ill patients. During convalescence, more detailed assessment of muscle strength, weakness, and physical function will provide better understanding in the trajectory of recovery.

## IMPACT ON CLINICAL OUTCOMES

As the impact of preexisting sarcopenia or frailty and clinical outcomes has been the focus of numerous studies in the past two years, we will only discuss meta-analysis and large cohort studies.

Preexisting sarcopenia was found in 43% of 3582 mechanically ventilated patients in a meta-analysis using the authors’ definitions from the original studies, which were heterogeneous and mostly based on low muscle mass observed on a CT-scan [1]. Only two studies included muscle strength into the definition. Sarcopenia was associated with increased mortality rates, and longer duration of mechanical ventilation, ICU- and hospital length of stay. In addition, preexisting frailty (CFS > 4) or malnutrition were also shown to be independently associated with increased acute skeletal muscle loss in ICU patients [18].

Furthermore, preexisting frailty (CFS > 4) was associated with increased short- and long-term mortality in both in-hospital and out-of-hospital cardiac arrest (IHCA and OHCA) [20,21]. Frail IHCA patients had a median survival of 19 days (vs. 302 days) and 30-days-survival in frail OHCA patients was 19.1% (vs. 39.2%). Each unit of increment in CFS was associated with 22% worse survival outcome in IHCA patients [20]. Favorable neurologic outcomes (CPC 1 or 2) were seen less in frail OHCA patients (6.1% vs. 24.4%) [21].

The effects of preoperative sarcopenia and frailty in critically ill patients on clinical outcomes was investigated in two recent large meta-analyses [22,23]. Nine different tools were used by the included studies to determine frailty, including unidimensional (focusing on physical performance) and multidimensional tools [22]. A significantly increased risk of short- and long-term mortality was found in frail (relative risk (RR): 2.66 and RR: 2.75) and sarcopenic patients, as well as an increase in ventilation duration, ICU- and hospital length of stay [17,18]. Frail patients were also more likely to be discharged to a healthcare facility [22]. An increase in postoperative complications was reported in sarcopenic patients [23]. A third meta-analysis on sarcopenic cardiac surgery patients reported similar effects on clinical outcomes, including an increased discharge to healthcare facilities [24].

Additionally, in an observational cohort of 293 elderly trauma patients (>65 years) frail and nonfrail patients were compared [25]. Ground-level fall was the predominant mechanism of injury in frail patients. However, traumatic brain injury was also more frequent in frail patients. At 6 months after the accident, 64.7% of frail patients had died (vs. 23.7%), 11.7% (vs. 20.5%) were hospitalized, 11.7% (vs. 1%) were discharged to a nursing home and only 11.7% (vs. 39.2%) of frail patients had returned home. Withholding or withdrawal of life support therapies were observed more frequently in frail patients.

Previous studies have focused on the effects of preexisting frailty on clinical outcomes; however, frailty severity may be dynamic as shown by the recent FORECAST study [26^▪▪^]. In a prospective multicenter observational study, 687 ICU patients ≥50 years old were included. Frailty was assessed by CFS and FI at ICU admission, hospital discharge, and 6 months. In survivors, frailty scores increased from ICU admission to hospital discharge but improved by 6 months.

## THERAPEUTIC INTERVENTIONS

Therapeutic trials specifically targeting sarcopenic or frail populations remain scarce. Given the shared focus of frailty and sarcopenia on diminished muscle strength and function, studies that aim to improve these parameters have been prioritized. Among these, nutrition and mobilization have been the most extensively studied interventions, with combination therapy trials gaining increasing attention in recent years.

### Nutrition

International guidelines provide no specific nutrition recommendations for patients with preexisting frailty or sarcopenia admitted to the ICU [27,28^▪^]. The recent PRECISe trial shows harm of high protein (2.0 g/kg/day) vs. standard protein (1.3 g/kg/day) isocaloric enteral nutrition (mean caloric intake of 19 kcal/kg/day in both groups) in mechanically ventilated ICU patients [29^▪▪^]. This effect is even stronger in women, which may be explained by a lower muscle mass in relation to body weight (i.e. lower fat free mass). Tailoring protein administration to muscle mass might prevent harm in patients with sarcopenia. Further studies are necessary to investigate the optimal protein dose, and its relation to muscle mass and function. In addition, an RCT of 60 patients revealed that tailoring energy targets by indirect calorimetry compared to predictive equations was associated with lesser calories fed, but also with lesser reduction in quadriceps muscle thickness in critically ill patients [30^▪^]. This once more underlines the harmful effects of overfeeding, of which patients with lower fat free mass may be at higher risk.

### Mobilization

Recent guidelines recommend protocol-based, step-wise mobilization for critical care patients within 72 h but are unable to give recommendations on duration of mobilization or contra-indications [31^▪^]. Early mobilization may be able to mitigate skeletal muscle loss, shorten ICU length of stay, and improve long-term functional outcomes, including cognitive function and quality of life [32–34,35^▪^]. Moreover, early mobilization has not been associated with an increase of (intervention-related) adverse events. However, no beneficial effects on mortality have been observed and high heterogeneity of studies limits conclusions on the effects of early mobilization on delirium [32,36].

The latest studies on mobilization interventions have explored a variety of approaches with mixed results. The ‘start-to-move’ mobilization protocol demonstrated promising results on functional outcomes but showed no effects on mortality, duration of mechanical ventilation, or ICU length of stay [37,38]. Neuromuscular electrostimulation has also garnered renewed attention, with reported positive effects on muscle mass, strength, and ICU-acquired weakness, though no impact on mortality [39]. Additionally, an RCT comparing early (<96 h) physical and occupational therapy with standard physiotherapy in 200 mechanically ventilated patients showed less cognitive impairment at hospital discharge (53.5 vs. 68.7%) and after one year (24.2% vs. 43.4%) in the intervention group. Higher physical component scores on quality-of-life testing, but no difference in functional independence were also found [35^▪^]. Further studies are required to gain more knowledge about the best type, intensity and duration of mobilization interventions.

### Rehabilitation and nutrition

Several pilot studies have explored the combination of various mobilization and nutrition strategies [40,41] (see more details in Table 1). However, the outcomes have been inconsistent, largely due to heterogeneous patient cohorts and significant variability in (standard) care practices. These findings highlight the need for further research to establish more consistent and evidence-based approaches.

**Table 1:** Pilot studies hat have explored the combination of various mobilization and nutrition strategies

Trials’ first author, year	Design	Outcome	Relevance/interpretation
Verceles, 2022 [40]	Randomized controlled trial of neuromuscular electrical stimulation 30 min twice daily for 14 days + high protein (1.75 g/kg/day) for 10 days + physiotherapy vs. standard care	39 patients included (I:16/C:23). Higher protein (1.3 vs. 0.8 g/kg/d), more PT sessions (+5.6) in intervention group. **Primary outcome:** Less muscle loss in intervention group at day 14, assessed by muscle volume and cross-sectional area **Secondary outcomes:** No difference in ICU LOS, duration of ventilation, nor proportion of patients who went home or to a nursing facility	Combination of neuromuscular electrical stimulation, protein supplementation and mobilization mitigated lower extremity muscle loss
Kagan, 2022 [41]	Three-arm, randomized controlled trial in ventilated ICU patients: -g1: conventional physiotherapy + standard EN (control) -g2: cycle ergometry 1d 20 min + standard EN -g3: cycle ergometry 1 day 20 min+ high protein EN until day 28, ICU discharge or death	62 patients included (22/21/19). Higher protein in g3 (83.7 vs. 63.6 and 67.2 g/day) **Primary outcome** No significant differences between groups in duration of mechanical ventilation. **Secondary outcomes:** No significant differences between groups for any of the secondary outcomes: ICU mortality, ICU LOS, hospital LOS, re-intubation rate	No beneficial effects of cycle ergometry with or without increased protein provision on clinical outcomes
de Azevedo, 2021 [42]	Randomized controlled trial in ventilated patients of high protein (2.0–2.2 g/kg/d) + early exercise (cycle ergometry 2 days 15 min) vs. control (protein: 1.4–1.5 g/kg/day) until day 21, discharge or death	181 patients included (I: 87/C: 94). No differences between calories received, higher protein in intervention group (1.48 vs 1.19 g/kg/day). **Primary outcome:** higher physical component summary score at 3 and 6 months in intervention group **Secondary outcomes:** lower ICU, hospital and 6-month mortality in intervention group. No difference in ICU or hospital LOS	Beneficial effect of protein & early mobilization on functional outcomes & mortality
Wu, 2023 [43]	Four-arm, single-blind randomized controlled trial of nonsurgical ICU patients: -g1: resistance training 20–30 min 5 days/week -g2: HMB 2 days 1.5 g -g3: resistance training 20–30 min 5 days/week + HMB 2 days 1.5 g -g4: control Until hospital discharge	112 patients included (25/28/24/24) **Primary outcome:** Increase in short physical performance battery and 6-min walking distance in g1 and g3 compared to control. No significant changes in g2 vs. control **Secondary outcomes:** Increased grip strength and MRC scores in g1 and g3 compared to control. No differences in mortality, length of stay	Resistance training and resistance training combined with HMB, but not HMB alone improved functional outcomes. No effects on mortality or length of stay
Zhou, 2022 [34]	Three-group randomized controlled trial of conscious ICU patients: -g1: early (<24 h), individualized, progressive mobilization 2 days 20–30 min -g2: early individualized progressive mobilization (<24 h) 2 days 20–30 min + early nutrition (<48 h) -g3: standard care Interventions until ICU discharge	150 patients included (50/50/50). **Primary outcome:** Significantly lower incidence of ICU-AW at ICU-discharge (MRC-sum < 48) in intervention groups (g1: 2%, g2: 2%, g3 16%). **Secondary outcomes:** Signifcantly better Barthel index, decreased length of ICU stay in the intervention groups. No differences in delta SOFA score, duration of ventilation, ICU mortality	Early mobilization, with or without early nutrition was associated with a lower incidence of ICU-AW at ICU-discharge
Elizabeth, 2024 [44]	Single-blind randomized, controlled pilot trial in ICU patients comparing indirected calorimetry directed feeding and 60 min cycle ergometry daily for 14 days with standard care (weight-based empiric feeding and physiotherapy)	21 patients included (11/10) **Primary outcome:** no significant differences in quadriceps muscle mass decrease (1.8 cm^2^ and 0.1 cm^2^ vs. 4.7 cm^2^ and 1.9 cm^2^ in controls)	Although not statistically significant, a trend towards decreased muscle loss in the combined intervention group was found

## CONCLUSION

The interplay between sarcopenia, frailty, and critical illness outcomes underscores the need for a comprehensive, multidisciplinary approach to care. Both conditions significantly influence patient's mortality, functional decline, and quality of life, persisting well beyond the ICU stay. Preexisting frailty negatively impacts clinical and functional outcomes in ICU patients, regardless of the reason for admission or the diagnostic tools used, which are notably heterogeneous. Importantly, assessing frailty prior to ICU admission provides valuable insights into survival chances and functional decline, supporting treatment decisions, advanced care planning, and end-of-life discussions.

Although functional outcomes are often reported indirectly, such as through rates of nursing home admission or increased daily care needs, they play a crucial role in the quality of life of ICU survivors. Mitigating functional decline should therefore be a key treatment goal. Frailty's dynamic nature - marked by transitions in severity during and after critical illness - highlights the opportunity for targeted interventions to attenuate adverse outcomes.

The substantial body of research conducted in the last years is encouraging, highlighting the growing awareness and attention that sarcopenia and frailty rightly deserve. However, significant heterogeneity stems not only from the diverse characteristics of these patient populations but also from the variability in diagnostic tools and criteria used to define these conditions. The limited number of studies specifically targeting interventions for sarcopenia and frailty in critical care populations highlight the need for further focused research in this area.

Tailored treatment strategies, rather than one-size-fits-all approaches, are likely to improve outcomes by addressing patient heterogeneity and aligning interventions with specific recovery goals. However, the current evidence is insufficient to recommend precise treatments for subgroups, emphasizing the need for further studies. Addressing sarcopenia and frailty within the intensive care unit requires a shift from generalized approaches to multidisciplinary, individualized strategies that align care with specific patient needs and goals. By doing so, healthcare providers can better support recovery, optimize functional outcomes, and improve overall quality of life for this vulnerable patient group after critical illness.

## Acknowledgements

*None.*

### Financial support and sponsorship

*None.*

### Conflicts of interest

*There are no conflicts of interest.*

## REFERENCES AND RECOMMENDED READING

Papers of particular interest, published within the annual period of review, have been highlighted as:

▪ of special interest
▪▪ of outstanding interest

## References

[1] Jiang T Lin T Shu X. Prevalence and prognostic value of preexisting sarcopenia in patients with mechanical ventilation: a systematic review and meta-analysis. Crit Care 2022; 26:140. 35578299 10.1186/s13054-022-04015-y PMC9109453

[2] Gustafson OD Williams MA McKechnie S. Musculoskeletal complications following critical illness: a scoping review. J Crit Care 2021; 66:60–66. 34454181 10.1016/j.jcrc.2021.08.002 PMC8516358

[3] Doody P Lord JM Greig CA. Frailty: pathophysiology, theoretical and operational definition(s), impact, prevalence, management and prevention, in an increasingly economically developed and ageing world. Gerontology 2023; 69:927–945. 36476630 10.1159/000528561 PMC10568610

[4] Westbury LD Beaudart C Bruyere O. Recent sarcopenia definitions-prevalence, agreement and mortality associations among men: Findings from population-based cohorts. J Cachexia Sarcopenia Muscle 2023; 14:565–575. 36604970 10.1002/jcsm.13160 PMC9891989

[5] Bhasin S Travison TG Manini TM. Sarcopenia definition: the position statements of the sarcopenia definition and outcomes consortium. J Am Geriatr Soc 2020; 68:1410–1418. 32150289 10.1111/jgs.16372 PMC12132920

[6] Cruz-Jentoft AJ Bahat G Bauer J. Sarcopenia: revised European consensus on definition and diagnosis. Age Ageing 2019; 48:601. 10.1093/ageing/afz046 PMC6593317 31081853

[7] Hamaker M Gijzel S Rostoft S. Intrinsic capacity and resilience: taking frailty to the next level. J Geriatr Oncol 2023; 14:101421. 36657249 10.1016/j.jgo.2022.101421

[8] Donini LM Busetto L Bischoff SC. Definition and diagnostic criteria for sarcopenic obesity: ESPEN and EASO consensus statement. Clin Nutr 2022; 41:990–1000. 35227529 10.1016/j.clnu.2021.11.014

[9] Wernly B Bruno RR Kelm M. Sex-specific outcome disparities in very old patients admitted to intensive care medicine: a propensity matched analysis. Sci Rep 2021; 11:14184. 34226660 10.1038/s41598-021-93448-6 PMC8257605

[10] Fried LP Tangen CM Walston J. Frailty in older adults: evidence for a phenotype. J Gerontol A Biol Sci Med Sci 2001; 56:M146–M156. 11253156 10.1093/gerona/56.3.m146

[11] Mitnitski AB Mogilner AJ Rockwood K. Accumulation of deficits as a proxy measure of aging. ScientificWorldJournal 2001; 1:323–336. 12806071 10.1100/tsw.2001.58 PMC6084020

[12] Rockwood K Song X MacKnight C. A global clinical measure of fitness and frailty in elderly people. CMAJ 2005; 173:489–495. 16129869 10.1503/cmaj.050051 PMC1188185

[13] Peters LL Boter H Burgerhof JG. Construct validity of the Groningen Frailty Indicator established in a large sample of home-dwelling elderly persons: evidence of stability across age and gender. Exp Gerontol 2015; 69:129–141. 25981741 10.1016/j.exger.2015.05.006

[14] Morley JE Malmstrom TK Miller DK. A simple frailty questionnaire (FRAIL) predicts outcomes in middle aged African Americans. J Nutr Health Aging 2012; 16:601–608. 22836700 10.1007/s12603-012-0084-2 PMC4515112

[15] Velanovich V Antoine H Swartz A. Accumulating deficits model of frailty and postoperative mortality and morbidity: its application to a national database. J Surg Res 2013; 183:104–110. 23415494 10.1016/j.jss.2013.01.021

[16] Bertschi D Waskowski J Schilling M. Methods of assessing frailty in the critically ill: a systematic review of the current literature. Gerontology 2022; 68:1321–1349. 35339999 10.1159/000523674 PMC9808663

[17] Falk Erhag H Guethnadottir G Alfredsson J. The association between the clinical frailty scale and adverse health outcomes in older adults in acute clinical settings – a systematic review of the literature. Clin Interv Aging 2023; 18:249–261. 36843633 10.2147/CIA.S388160 PMC9946013

[18] Kangalgil M Kucuk AO Ulusoy H. Nutrition determinants of acute skeletal muscle loss in critically ill patients: a prospective observational cohort study. Nutr Clin Pract 2024; 39:579–588. 37877164 10.1002/ncp.11086

[19] Sundarsingh V Manoj Kumar R Kulkarni M. Quadriceps muscle layer thickness and its association with frailty in critically ill patients: a prospective observational study. J Crit Care 2024; 85:154930. 39426182 10.1016/j.jcrc.2024.154930

[20] Ueno R Reddy MP Jones D. The impact of frailty on survival times up to one year among patients admitted to ICU with in-hospital cardiac arrest. J Crit Care 2024; 83:154842. 38865757 10.1016/j.jcrc.2024.154842

[21] Yamamoto R Tamura T Haiden A. Frailty and neurologic outcomes of patients resuscitated from nontraumatic out-of-hospital cardiac arrest: a prospective observational study. Ann Emerg Med 2023; 82:84–93. 36964008 10.1016/j.annemergmed.2023.02.009

[22] Chan R Ueno R Afroz A. Association between frailty and clinical outcomes in surgical patients admitted to intensive care units: a systematic review and meta-analysis. Br J Anaesth 2022; 128:258–271. 34924178 10.1016/j.bja.2021.11.018

[23] Yang TR Luo K Deng X. Effect of sarcopenia in predicting postoperative mortality in emergency laparotomy: a systematic review and meta-analysis. World J Emerg Surg 2022; 17:36. 35752855 10.1186/s13017-022-00440-0 PMC9233792

[24] Ansaripour A Arjomandi Rad A Koulouroudias M. Sarcopenia adversely affects outcomes following cardiac surgery: a systematic review and meta-analysis. J Clin Med 2023; 12:5573. 37685640 10.3390/jcm12175573 PMC10488406

[25] Legros V Seube-Remy PA Floch T. Frailty and 6-month trajectory of elderly trauma patients over the age of 65 years admitted to intensive care unit for severe trauma: experience of a level 1 trauma center. BMC Geriatr 2024; 24:759. 39277744 10.1186/s12877-024-05350-1 PMC11401312

[26▪▪] Muscedere J Bagshaw SM Kho M. Frailty, outcomes, recovery and care steps of critically ill patients (FORECAST): a prospective, multicentre, cohort study. Intensive Care Med 2024; 50:1064–1074. 38748266 10.1007/s00134-024-07404-9 PMC11245420

[27] Compher C Bingham AL McCall M. Guidelines for the provision of nutrition support therapy in the adult critically ill patient: The American Society for Parenteral and Enteral Nutrition. JPEN J Parenter Enteral Nutr 2022; 46:12–41. 34784064 10.1002/jpen.2267

[28▪] Singer P Blaser AR Berger MM. ESPEN practical and partially revised guideline: clinical nutrition in the intensive care unit. Clin Nutr 2023; 42:1671–1689. 37517372 10.1016/j.clnu.2023.07.011

[29▪▪] Bels JLM Thiessen S van Gassel RJJ. Effect of high versus standard protein provision on functional recovery in people with critical illness (PRECISe): an investigator-initiated, double-blinded, multicentre, parallel-group, randomised controlled trial in Belgium and the Netherlands. Lancet 2024; 404:659–669. 39153816 10.1016/S0140-6736(24)01304-7

[30▪] Chandrasekaran A Pal D Harne R. Comparison between effect of indirect calorimetry vs weight-based equation (25 kcal/kg/day)-guided nutrition on quadriceps muscle thickness as assessed by bedside ultrasonography in medical intensive care unit patients: a randomized clinical trial. Indian J Crit Care Med 2024; 28:587–594. 39130394 10.5005/jp-journals-10071-24737 PMC11310671

[31▪] Schaller SJ Scheffenbichler FT Bein T. Guideline on positioning and early mobilisation in the critically ill by an expert panel. Intensive Care Med 2024; 50:1211–1227. 39073582 10.1007/s00134-024-07532-2

[32] Matsuoka A Yoshihiro S Shida H. Effects of mobilization within 72 h of ICU admission in critically ill patients: an updated systematic review and meta-analysis of randomized controlled trials. J Clin Med 2023; 12:58888. 10.3390/jcm12185888 PMC10531519 37762829

[33] Zang K Chen B Wang M. The effect of early mobilization in critically ill patients: a meta-analysis. Nurs Crit Care 2020; 25:360–367. 31219229 10.1111/nicc.12455

[34] Zhou W Yu L Fan Y. Effect of early mobilization combined with early nutrition on acquired weakness in critically ill patients (EMAS): a dual-center, randomized controlled trial. PLoS One 2022; 17:e0268599. 35617287 10.1371/journal.pone.0268599 PMC9135241

[35▪] Patel BK Wolfe KS Patel SB. Effect of early mobilisation on long-term cognitive impairment in critical illness in the USA: a randomised controlled trial. Lancet Respir Med 2023; 11:563–572. 36693400 10.1016/S2213-2600(22)00489-1 PMC10238598

[36] Nydahl P Jeitziner MM Vater V. Early mobilisation for prevention and treatment of delirium in critically ill patients: systematic review and meta-analysis. Intensive Crit Care Nurs 2023; 74:103334. 37440187 10.1016/j.iccn.2022.103334

[37] Hoffman M Clerckx B Janssen K. Early mobilization in clinical practice: the reliability and feasibility of the ‘Start To Move’ protocol. Physiother Theory Pract 2022; 38:908–918. 32866055 10.1080/09593985.2020.1805833

[38] Soto S Adasme R Vivanco P. Efficacy of the ‘start to move’ protocol on functionality, ICU-acquired weakness and delirium: a randomized clinical trial. Med Intensiva (Engl Ed) 2024; 48:211–219. 38402053 10.1016/j.medine.2024.01.003

[39] Nakanishi N Yoshihiro S Kawamura Y. Effect of neuromuscular electrical stimulation in patients with critical illness: an updated systematic review and meta-analysis of randomized controlled trials. Crit Care Med 2023; 51:1386–1396. 37232695 10.1097/CCM.0000000000005941

[40] Verceles AC Serra M Davis D. Combining exercise, protein supplementation and electric stimulation to mitigate muscle wasting and improve outcomes for survivors of critical illness – the ExPrES study. Heart Lung 2023; 58:229–235. 36473808 10.1016/j.hrtlng.2022.11.013 PMC9992240

[41] Kagan I Cohen J Bendavid I. Effect of combined protein-enriched enteral nutrition and early cycle ergometry in mechanically ventilated critically ill patients – a pilot study. Nutrients 2022; 14:1589. 35458151 10.3390/nu14081589 PMC9027967

[42] de Azevedo JRA Lima HCM Frota P. High-protein intake and early exercise in adult intensive care patients: a prospective, randomized controlled trial to evaluate the impact on functional outcomes. BMC Anesthesiol 2021; 21:283. 34773985 10.1186/s12871-021-01492-6 PMC8590269

[43] Wu TT Chen QL Lin XX. Effects of a multilevel intervention of resistance training with or without beta-hydroxy-beta-methylbutyrate in medical ICU patients during entire hospitalisation: a four-arm multicentre randomised controlled trial. Crit Care 2023; 27:493. 38102705 10.1186/s13054-023-04698-x PMC10724983

[44] Elizabeth NSH Yanni T May LS. Indirect calorimetry directed feeding and cycling in the older ICU population: a pilot randomised controlled trial. BMC Anesthesiol 2024; 24:171. 38714926 10.1186/s12871-024-02507-8 PMC11075192

